# The Cure of Rupture, Reducible and Irreducible; Also of Variocele and Hydrocele, by New Methods

**Published:** 1877-06

**Authors:** 


### BIBLIOGRAPHICAL.

The Cure of Rupture, Reducible and Irreducible; also of Varicocele and Hydrocele, by New Methods. By George Heaton, M.D., member Massachusetts Medical Society; Fellow of the Royal Chirurgical Society of London; of the Westminster and London Medical Society; of the Parisian Medical Society of France, etc. Arranged and edited by J. Henry Davenport, A.M., M.D., Harv. H. O. Houghton & Co., publishers. Boston. 1877.

The author claims in this to have done away with the unsatisfactory and dangerous procedures usually resorted to for the radical cure of hernia. Heretofore, in all cases in which this radical cure has been attempted, he claims that the ephemeral benefit obtained is in no wise comparable to the difficulties and dangers incurred.

The truth of this we are constrained to acknowledge, and could it be established that this treatment advised by the author is really calculated to prove satisfactory, and capable of modifying a heretofore dangerous undertaking into an innocent procedure, an inestimable boon will have been conferred on mankind.

The results promised by his manner of treatment are worthy, we should think, an investigation, and we therefore recommend the work to the consideration of those wishing to test its merits.

